# Systematic Review of Nano- and Microplastics’ (NMP) Influence on the Bioaccumulation of Environmental Contaminants: Part II—Freshwater Organisms

**DOI:** 10.3390/toxics11060474

**Published:** 2023-05-23

**Authors:** Fabianne Ribeiro, Maria D. Pavlaki, Susana Loureiro, Renato Almeida Sarmento, Amadeu M. V. M. Soares, Paula S. Tourinho

**Affiliations:** 1 CESAM—Centre for Environmental and Marine Studies, Department of Biology, University of Aveiro, Campus Universitário de Santiago, 3810-193 Aveiro, Portugal; 2 Programa de Pós-Graduação em Produção Vegetal, Universidade Federal do Tocantins, Campus de Gurupi, Gurupi 77402-970, TO, Brazil; 3 Department of Environmental Chemistry, Faculty of Environmental Technology, University of Chemistry and Technology Prague, Technická 5, 166 28 Prague, Czech Republic

**Keywords:** bioaccumulation, microplastics, nanoplastics, carrier role, freshwater

## Abstract

Nano- and microplastic fragments (NMPs) exist ubiquitously in all environmental compartments. The literature-based evidence suggests that NMPs interact with other environmental contaminants in freshwater ecosystems through sorption mechanisms, thereby playing a vector role. Chemically bound NMPs can translocate throughout the environment, reaching long distances from the contaminant discharge site. In addition, they can be ab/adsorbed by freshwater organisms. Although many studies show that NMPs can increase toxicity towards freshwater biota through the carrier role, little is known regarding their potential to influence the bioaccumulation of environmental contaminants (EC) in freshwater species. This review is part II of a systematic literature review regarding the influence of NMPs on bioaccumulation. Part I deals with terrestrial organisms and part II is devoted to freshwater organisms. The Preferred Reporting Items for Systematic Reviews and Meta-Analyses Extension for Scoping Reviews (PRISMA ScR) was used for the literature search and selection. Only studies that assessed the bioaccumulation of EC in the presence of NMPs and compared this with the bioaccumulation of the isolated EC were considered. Here, we discuss the outcome of 46 papers, considering NMPs that induced an increase, induced a decrease, or caused no effect on bioaccumulation. Lastly, knowledge gaps are identified, and future directives for this area of research are discussed.

## 1. Introduction

Nano- and microplastics (NMPs) are small particulate plastic fragments ubiquitously present in the environment [1]. They are considered emerging contaminants due to their wide distribution, small size, environmental persistence, and ability to interact with other environmental contaminants (ECs) of concern, which may, in some cases, drive an exacerbated toxicity of ECs on biota [2,3,4,5]. Two main mechanisms are accountable for the alteration of toxicity when microplastics are combined with other ECs. First, sorption/desorption [6,7,8,9,10] of substances onto the NMPs’ surface can facilitate contaminant internalization by organisms via, e.g., feeding on the NMPs [11,12]. Second, NMPs alone can induce physical damage to specific tissues, including mechanical aggravation of biological barriers, such as membranes, skin, cell walls and gills, thereby increasing the organism’s permeability and susceptibility to ECs [13,14]. Moreover, the degree of interaction between NMPs and ECs is governed by the chemical characteristics of both components in this combination (e.g., the polymer composition and chemical nature of ECs). Notwithstanding, to comprehend the consequences of toxicity and potential bioaccumulation arriving from the combination of NMPs and ECs, one must bear in mind that the physiology and other traits of the studied species are the core variable in the resulting effects. The state-of-the-art concerning the ecotoxicity of diverse classes of ECs in combination with NMPs provides robust literature portraying numerous effects in freshwater, marine, and terrestrial species. However, the bioavailability of substances combined with NMPs is often neglected, considering the organism under study. Bioavailability is herein defined as the available fraction of a certain compound, which, when entering the biological system, will interact with its components and trigger adverse effects [15,16,17]. 

Some organisms frequently applied as model species (e.g., *Daphnia* spp., zebrafish, green unicellular algae) have intrinsic anatomic, physiological, and behavioural traits that will ultimately ensure that the absorption of NMPs takes place. For instance, *Daphnia*’s filtering appendages can unselectively internalize particles up to 80 μm in size [11], which includes bacteria, algae cells, and other particulate organic and inorganic (e.g., NMPs) matter within this size range [18]. Previous studies indicate that the external carapace of *Daphnia* sets the upper size limit for ingestion (80 μm) [11], whereas the mesh size of the filtering appendages outlines the lower limit for absorption (200 nm) [19]. In this case, if *Daphnia* is exposed to particles exceeding 80 μm, there is a low probability of the uptake of either the NMPs or the adsorbed ECs (not neglecting, however, the possible uptake of the dissolved substance that may occur via filtration or passive diffusion). In this scenario, the carrier role of NMPs cannot be assessed because NMPs are simply not bioavailable, and the observed effects (if any) caused by them will be mostly mechanical (e.g., obstruction of the filtering appendages) [20]. Although numerous studies exist regarding the influence of NMPs on the toxicity of the ECs of concern, the influence of NMPs on bioaccumulation is still poorly explored. 

As many authors associate the increased toxicity observed in combined exposures to the carrier role of NMPs [6,21], it is also important to understand how NMPs will influence the bioaccumulation of ECs, and if, upon continued exposures, the carrier role of NMPs persists. 

Based on the above, the present review aims at answering the following question: ow does the presence of NMPs affect the bioaccumulation of contaminants? For this, the Preferred Reporting Items for Systematic Reviews and Meta-Analyses Extension for Scoping Reviews (PRISMA ScR) was followed to systematically search for evidence on the NMP–chemicals–bioaccumulation interaction and to identify knowledge gaps on this topic. Only publications that compared treatments with or without NMPs were considered as inclusion criteria. To make a systematic review as comprehensive as possible, the retrieved articles were separated into three main groups, namely terrestrial, freshwater, and marine, following the literature selection. In this review, we will focus on publications retrieved from the literature regarding the bioaccumulation of ECs in the presence of NMPs by freshwater species.

## 2. Materials and Methods

### 2.1. Search Strategy

This review followed the guidelines from the Preferred Reporting Items for Systematic Reviews and Meta-Analyses Extension for Scoping Reviews (PRISMA-ScR) [22]. A comprehensive search was performed in the Web of Science and Scopus databases, using the algorithm: (microplastic* OR nanoplastic*) AND ((toxic compound* OR pollutant* OR contaminant* OR hydrophobic organic contaminant* OR persistent organic contaminant* OR metals) AND (bioaccumulation OR accumulation OR desorption)) AND (environment* OR ecotoxic*). The search in both databases was conducted on June 29, 2021. The search retrieved 3601 scientific outputs, 995 from Web of Science and 2606 from Scopus. After an initial check, 422 duplicates were found. Afterwards, the title and abstract screening of 3179 articles was conducted.

### 2.2. Selection Process

An inclusion criterion was used during the title and abstract screening, with only original research articles published in English being considered. Other publication types, such as reviews, books or book chapters, and conference papers, were not included. Moreover, studies that did not explicitly indicate joint exposure to nano/microplastics and contaminants or the use of environmentally important freshwater species (i.e., animal models used in medical studies) were excluded. The studies included in the final selection had their data extracted into a table in Excel (Supplementary Information). Information on the study characteristics included: (1) exposure route (water, diet, or both); (2) spiking procedure; (3) nano/microplastic characteristics (size, form, and polymer type); (4) contaminant’s name and chemical group; (5) species’ name and group; and, finally, (6) the outcome (i.e., whether nano/microplastic influenced bioaccumulation). For the spiking procedure categorization, three methods were considered: pre-incubation, where NMPs were previously incubated (e.g., 24 h, 48 h) with the contaminant solution before exposure; pre-contamination, where NMPs were contaminated immediately before being added to the exposure medium; and freshly spiking, where NMPs and contaminants were added to the exposure medium immediately prior to exposure. In fish studies, bioaccumulation was also compared between the developmental stages (larvae and adults).

## 3. Results

### 3.1. Literature Search and Selection

**Figure 1:**
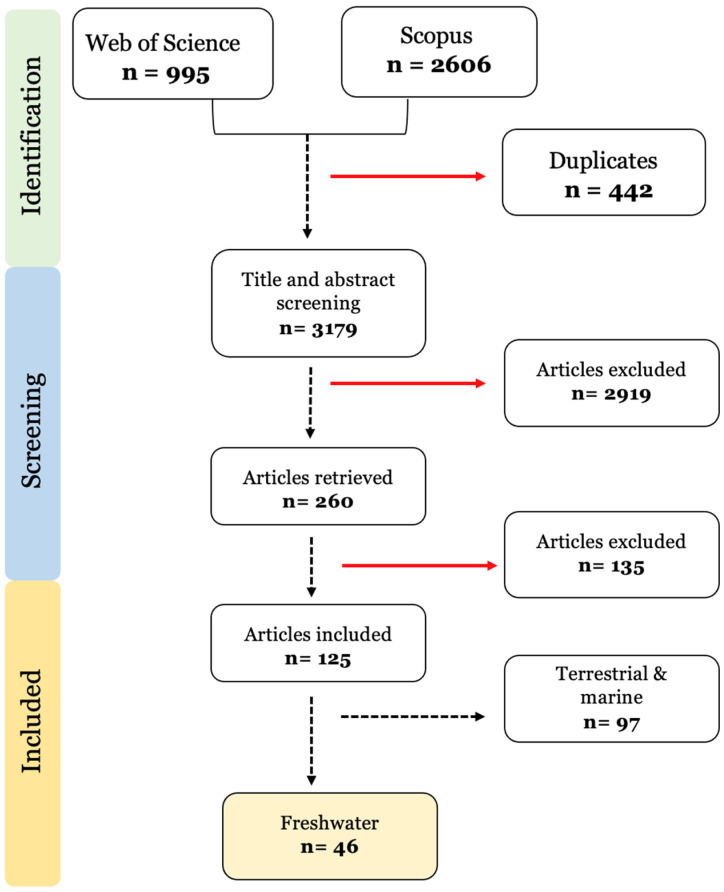
Prisma diagram of the selection method and exclusion criteria used in the systematic review. n = number of studies.

The number of publications considered in each phase of the literature search and selection can be found in Figure 1. After the manual title and abstract screening of 3179 articles, 260 articles were identified as relevant and had their full text analysed. In total, 135 further articles were excluded due to: (1) a lack of treatments exposed to contaminants alone to allow for comparison (n = 61); (2) no bioaccumulation quantification was assessed (n = 25); (3) field studies that did not assess the relationship between contaminant-body levels and microplastic exposure (n = 25); (4) desorption under gut simulated conditions (n = 20); (5) microplastic added only in the depuration phase (n = 3); and (6) insufficient sample size.

From the remaining 125 publications, the ones covering species from marine and terrestrial habitats were excluded. Finally, only 46 publications were included in this systematic review (Figure 1).

### 3.2. Characteristics of the Selected Studies

**Figure 2:**
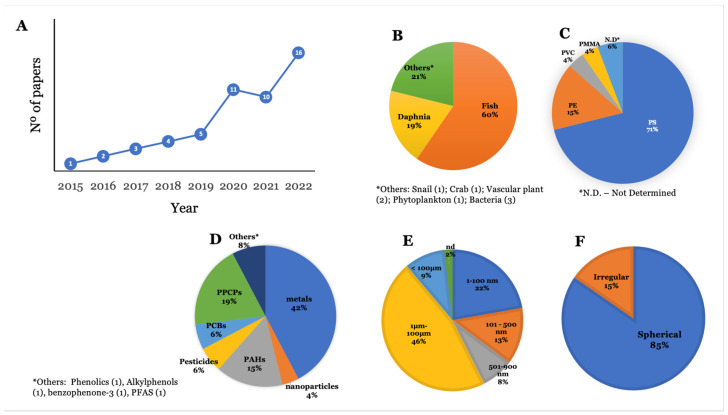
Number of selected publications by year published (**A**) and categorization of selected papers according to (**B**) group of organisms studied, (**C**) polymer type, (**D**) group of environmental contaminants, (**E**) size range, and (**F**) shape of NMP.

Figure 2A depicts the number of publications considered in this review per year of publication. It clearly shows an increase in the number of studies considering the influence of NMPs on the bioaccumulation of environmental contaminants. The advances in analytical chemistry and NMP characterization techniques have contributed to this increase regarding the quantification of contaminants in the animals’ tissue and exposure media [23,24,25]. Fish are the most studied group of animals within this subject (60%), with *Danio rerio* being the most studied species (Figure 2B). *Daphnia magna* is the second most used species to assess the influence of NMPs on the bioaccumulation of contaminants. Other species used were unicellular algae, freshwater crabs and snails, bacteria, protozoa, and vascular plants [26,27,28,29,30,31,32,33,34,35] (Figure 2B). As for the polymer type, polystyrene (PS) is the most studied polymer (70%), as it also stands as the third most abundant polymer in the environment, after polyethylene (PE) and polypropylene (PP) (Figure 2C) [36], which gives it an environmental relevance. Following PS, PE is the second most used polymer (15%) for investigating the influence of NMPs on the bioaccumulation of contaminants. Other types of polymers used were poly(methyl methacrylate) (PMMA) and polyvinyl chloride (PVC), whereas 6% of the studies did not specify the type of polymer applied (Figure 2C).

Considering the chemical group of environmental contaminants, metals were the most studied group (42%), followed by pharmaceuticals and personal care products (PPCPs) (19%) (Figure 2D). Regarding the route of exposure, 98% of studies performed the joint exposure of NMPs and other substances via water. Only one paper assessed exposure via water and food [37], and one study employed sediment exposure [34]. Regarding NMP size, 9% of studies employed NMPs larger than 100 μm, 46% of experiments used NMPs in the range of 1 to 100 μm, 13% of studies used NMPs between 101 nm and 500 nm, 22% employed NMPs with sizes between 1 nm and 100 nm, 8% of studies employed NMPs with sizes ranging from 500 nm to 900 nm, and 2% of the analysed studies did not mention the size of the NMPs used (Figure 2E). Regarding shape, most experiments used spherical NMPs (85%), whereas irregular NMPs were used in 15% of the studies (Figure 2F). For a more comprehensive discussion of results, this review is divided into the influence of NMPs on the bioaccumulation of contaminants by groups of organisms retrieved during the literature search, as shown in Figure 2B. 

### 3.3. Studies with Fish

**Figure 3:**
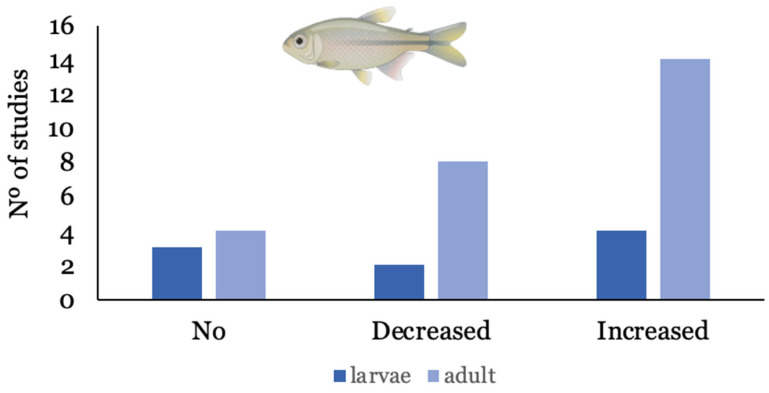
Number of studies where bioaccumulation of ECs was increased, decreased, or suffered no influence, according to the developmental stage of fish.

Out of the 29 studies considered, *Danio rerio* (zebrafish) is the most studied species, presented in 70% of the studies that employed fish as a model species. Other species used to assess the influence of NMPs on the bioaccumulation of contaminants were *Carassius auratus* (n = 1) [38], *Oreochromis niloticus* (n = 3), [39,40,41], *Misgurnus anguillicaudatus* (n = 1) [42], *Symphysodon aequifasciatus* (n = 1) [43], and *Cyprinus carpio* (n = 1) [44], and one study used in vitro gut sacs of rainbow trout *Oncorhynchus mykiss* [45]. In all studies with fish, water was the exposure route used, except for one study that employed dietary exposure to assess the trophic transfer of benzo(k)fluoranthene (bkf) [37]. Metals were the most studied group of contaminants (45%) and, among organic compounds, PPCPs were presented in most studies (25%). In half of the experiments (54%), the presence of NMPs increased the bioaccumulation of other contaminants, while in 22.5% of studies, NMPs decreased bioaccumulation; 25% indicated that the presence of NMPs did not influence bioaccumulation. Overall, the presence of NMPs increased the bioaccumulation of organic compounds and metals in fish in 58% and 42% of studies, respectively. There was also a tendency for a developmentally related influence of NMPs on bioaccumulation, as shown in Figure 3, where the decrease and increase are most often observed in adult fish, in relation to larvae.

### 3.4. Studies with Bacteria, Algae, and Macrophyte

Nine studies were retrieved from the literature which considered the influence of NMPs on the bioaccumulation of contaminants in zooplankton species. All experiments were conducted with *Daphnia magna* as a model species (except for one study that used *Daphnia magna* and *Chironomus riparius* [37]). Half of the considered studies assessed the bioaccumulation of PAHs in Daphnia [17,46,47], while 30% reported the bioaccumulation of metals [48,49], and the other 20% reported the bioaccumulation of benzophenone-3 and PCB [2,50]. 

Two studies explored the role of NMPs in the bioaccumulation of ECs in microorganisms, by employing the bacteria *Escherichia coli* [32] and *Streptomyces coelicolor* [28] and the protozoa *Euglena gracilis* [27] as model organisms. One study assessed the bioaccumulation of ibuprofen in the presence of NMPs on *Chlorella pyrenoidosa* [33], and two studies evaluated the influence of NMPs in the bioaccumulation of ECs by macrophytes Potamogeton crispus and *Vallisneria denseserrulata* and *Vallisneria natans* (Lour) [26,34]. 

## 4. Discussion

This review aimed to disentangle whether the presence of NMPs influences the bioaccumulation of environmental contaminants in freshwater species. After a systematic search of the literature, where the current review showed a small variability in the studied species (mostly focused on *Danio rerio* and *Daphnia magna*), the outcome of bioaccumulation was not unanimous, i.e., the presence of NMPs did not consistently influence bioaccumulation of the studied compounds. While there are several reasons for the inconsistency observed in the results (with the physiology of the tested species playing an important role), here, we will focus on the main factors that make the presence of NMPs influence bioaccumulation, such as the chemical class of contaminants and its interaction with the NMPs, the uptake route, and polymer size, among others. Hereafter, a particle size of up to 1 μm (i.e., 1000 nm) will be considered a nanoplastic (NP), whereas particles larger than 1 μm will be considered microplastics (MPs) [51]. 

For informative purposes, this discussion will be divided between the two main biological groups tested: fish and other species (including bacteria, protozoa, crab, bivalve, macrophytes, and zooplankton).

### 4.1. Influence of NMPs on the Bioaccumulation of Environmental Contaminants in Fish

#### 4.1.1. Increase in Bioaccumulation Induced by NMPs

**Figure 4:**
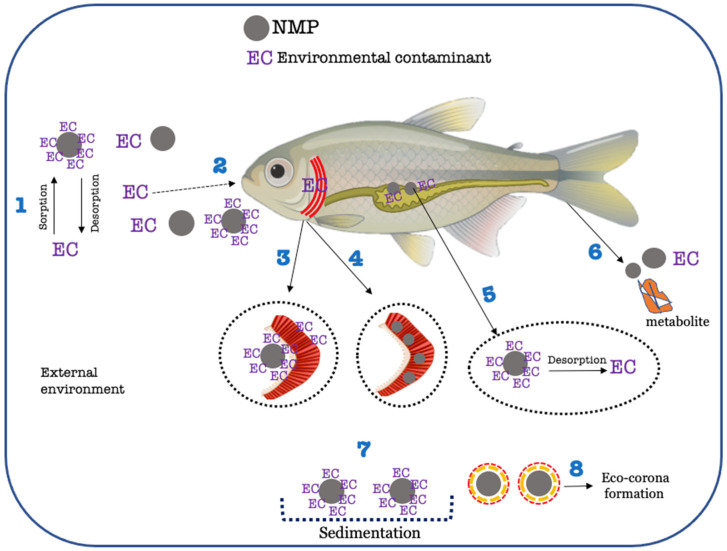
Simplified scheme of possible interactions between environmental contaminants (ECs) and NMPs in a freshwater environment and within a fish organism. 1—Sorption/desorption of EC onto NMPs. 2—Oral uptake (via water or food) of ECs, NMPs, and ECs adsorbed onto NMPs. 3—Uptake of ECs via gills (either dissolved or adsorbed onto NMPs, carrier effect). The size of NMPs is important to whether NMP internalization will occur. For larger particles, bioavailability will decrease, and direct uptake through gills will be lower. 4—Uptake of NMPs via gills. In this case, NMPs clothe gill membranes, decreasing the uptake of ECs. 5—Sorption/desorption of ECs onto NMPs in the gastrointestinal tract after oral ingestion. The internal environment of the gastrointestinal tract can favour the desorption of contaminants and their blood distribution through the body. 6—Excretion/Egestion of ECs and/or metabolites favoured by the presence of NMPs. This is one of the explanations for lowered bioaccumulation of ECs in the presence of NMPs. 7—Sedimentation of NMPs loaded with ECs. In this case, the EC becomes less available, and uptake and bioaccumulation of the substance decrease. 8—Formation of a layer of organic matter around the NMPs, designated by Eco-corona. Eco-corona formation may induce faster sedimentation, impacting environmental fate and bioavailability of NMPs.

Freshwater fish are exposed to NMPs via the respiratory tract (e.g., gills), by accidentally swallowing polymer particles mistaken for food and by feeding on prey that carry NMPs in their bodies [52]. Therefore, for NMPs to increase the uptake of a certain contaminant in fish, the contaminant should either be adsorbed to the particle being ingested or taken up in the fish gills due to respiration (Figure 4). Apart from two experiments that assessed the trophic transfer and bioaccumulation of contaminants in the presence of NMPs through food only [37,38], more than 90% of those discussed within this literature review were performed by exposing fish to contaminants and NMPs via water. Among experiments with exposure via water, 25% of them tested polystyrene nanoplastics (PS-NPs, ≤100 nm) in combination with other contaminants. The environmental contaminants studied in combination with PS-NPs were copper (Cu^+2^), [40,53], aluminium (nAl_2_O_3_) and cerium oxide nanoparticles (nCeO_2_) [54], bisphenol-A (BPA) [55], roxithromycin (ROX) [41], and the UV-filter ethylhexyl salicylate (EHS) [56]. In all cases, PS-NPs increased the bioaccumulation of contaminants, regardless of whether the pre-incubation or pre-sorption approach was performed. This is possibly linked to the size of NPs (≤100 nm), where smaller particles such as nanoplastics have a great surface area to volume ratio, which increases their adsorption capacity and, consequently, the uptake and accumulation of sorbed substances. Furthermore, nano-scaled fragments are more prone to enter the biological barriers of fish, such as gills and intestine [57]. As shown by the study of Qiao et al. (2019) [53], Cu^+2^ concentration in zebrafish was ~2-fold higher in the small particle exposure (≤100 nm) when compared to the larger particle exposure (20 μm). In addition, nano-scaled particles are less prone to egestion, being trapped in the microvilli of the gut once uptake occurs [39]. Moreover, in cases where the concentration of one of the components in the combination (NMPs or EC) varied, the EC accumulation was increased at the higher exposure doses. With the same PS-NP concentration (1 mg/L) combined with 0.5 mg/L, 1 mg/L, and 2 mg/L Cu^+2^, Nile tilapia *Oreochromis niloticus* accumulated higher amounts of Cu^+2^ in the liver, with increasing Cu^+2^ concentrations in the water [40]. In the study of Zhang et al. (2019) [41], the accumulation of ROX was higher with increasing concentrations of PS-NPs, for the same concentration of ROX in solution. Those findings suggest that concentration (either of NMPs or contaminants under study) is a major factor influencing bioaccumulation. It also appears that concentration of both NMPs and ECs dictates the level of interaction between NMPs and dissolved substances (sorption/desorption mechanisms) and, in turn, with fish [6,7] (Figure 4). Regarding experiments that explored the effects of larger particles (0.1–500 μm) on the bioaccumulation of contaminants in fish, their outcome was not unanimous concerning the influence of the NMPs. The presence of NMPs increased, decreased, and had no effect on the bioaccumulation of distinct classes of contaminants in fish. Again, several factors are associated with the discrepancy in the results reported by those studies. First, the increase in bioaccumulation driven by the presence of NMPs was observed for dissimilar chemical classes of contaminants, such as PPCPs [39,42,58], metals [53,59,60], metal nanoparticles [5], and PAHs [61,62]. In all cases, the NMPs acted as carriers for the contaminants. This implies that chemical affinity between the polymer type and the substance under study needs to occur. Moreover, the role of the polymers as carriers can be influenced by test variables, such as (i) the surface area of the particle and (ii) the concentration of the polymer. Aged PS-MPs showed an increase in the bioaccumulation of sulfamethoxazole (SMX) [39], when compared to pristine MPs, due to the alterations in their surface morphology, resulting in higher adsorption of SMX. In another study, the increased concentration of PVC (<10 μm) did not promote a higher clearance of Venlafaxine from water, possibly due to a higher rate of collisions between particles, which prevented adsorption [42]. Nevertheless, the bioconcentration factor (BAF) of Venlafaxine was 10-fold higher in fish subjected to joint exposure with PVC-MPs, compared to Venlafaxine alone, proving the carrier role of the PVC-MPs [17]. On the other hand, zebrafish exposed to cadmium (Cd) and PS-MPs accumulated higher amounts of Cd in the organs at the exposure, where PS-MPs were higher (200 μg/L, compared to 20 μg/L), while Cd concentration was kept constant (10 μg/L). This suggests, once again, that the chemical nature of both (the polymer and substance under study) will ultimately dictate the type of interaction between them and the degree to which MPs will influence the bioaccumulation in the organism.

#### 4.1.2. Decrease in Bioaccumulation Induced by NMPs

A decrease in the bioaccumulation of environmental contaminants in fish, induced by the presence of NMPs, was observed in a few studies [38,43,44,63,64,65,66]. Most exposures were performed via water, except for one experiment where exposure was conducted via food [38]. The NMPs studied were PE and PS with a large size range (5 to 500 μm), combined with a diverse class of environmental contaminants (PCBs, metals, pesticides, PFASs, and PPCPs). 

Experiments showing a decrease in uptake and bioconcentration of contaminants in fish linked this effect to a decrease in the bioavailable fraction of the EC, induced by the presence of NMPs. It is suggested that this decrease in bioavailability is due to the adsorption of the contaminants to the NMPs [43], which fish do not internalize. The most direct uptake routes in fish are the gills and skin, through which a substance can directly reach the bloodstream [67]. In cases where NMPs adsorbed with the substance are ingested, they must undergo digestion and metabolism before reaching the bloodstream. Furthermore, it is hard to predict the degree of desorption of the contaminant inside the gastrointestinal tract (it may vary widely depending on its chemical class and the polymer composition). Therefore, the bioavailability of a compound adsorbed to NMPs decreases once ingested. This was corroborated by the work of Schell et al. (2022) [65], where the uptake and bioconcentration of two hydrophobic organic chemicals (Chlorpyrifos (CPF) and Hexachlorobenzene (HCB)) by zebrafish were greatly decreased in the presence of PE-MPs. The authors suggested a direct uptake via gills representing a straighter exposure route of chemicals, caused by the greater bioavailability of the substance when it is not adsorbed to NMPs (i.e., dissolved in water). In the study of Changsheng Li et al. (2022) [64], it is also argued that the lowered internal concentration of difenoconazole (DIF) in zebrafish detected at the combined treatment (DIF (0.02 mg/L) + PS-MPs (1 mg/L)) was due to attraction forces between DIF and PS-MPs (adsorption) being stronger than the ones between DIF and the fish tissues (gills and skin). Moreover, the authors suggest that the adsorption of DIF onto PS-MPs could occur in the gastrointestinal lumen, thereby facilitating the egestion of DIF, which also contributes to the decrease in the internal concentration observed. 

Silver (Ag^+^), at an environmentally relevant concentration (1 μg/L), combined with polyethylene microplastics (PE-MPs) caused no effect on the uptake of Ag^+^ by zebrafish [63]. However, pre-sorption (where 75% of dissolved Ag^+^ was adsorbed to PE-MPs) decreased the overall uptake of Ag (lower bioavailability), and most of the internalized Ag^+^ was found in the intestine. 

#### 4.1.3. No Effect of NMPs on Bioaccumulation

In studies where the presence of NMPs did not affect the bioaccumulation of ECs in fish, the tested contaminants were from different chemical classes: PAHs [11], metals [45,68,69,70], pesticides [64], and PCBs [71]. Most exposures were performed via water except for one experiment in vitro [45] and one experiment via food (the final aim was to assess trophic transfer) [37]. There does not seem to be a common reason that explains the cases in which the presence of NMPs did not affect bioaccumulation. However, the evidence suggests that different interactions can lead to this outcome, whether in the external environment or within the organism. In the trophic transfer experiment [11], *Daphnia magna* and *Chironomus riparius* were pre-exposed to PMMA loaded with benzo(k)fluoranthene (BkF) and then used to feed adult zebrafish. Although the uptake and accumulation of BkF by *D. magna* and *C. riparius* were higher in the combined exposure, this did not translate into a higher concentration of BkF in adult zebrafish. The authors related this finding to the possibility of metabolic transformation of BkF by *D. magna* and *C. riparius*, which have been known to transform other PAHs metabolically. The resulting metabolites were less bioavailable to zebrafish, ultimately preventing trophic transfer. 

Zebrafish, fed for 7 days on MPs loaded with PCB, failed to accumulate higher amounts of PCB compared to PCB associated with food particles because fish stopped recognizing plastic particles as food and were no longer ingesting them [36]. In this case, the uptake route of PCB was restricted to gill uptake of the waterborne compound because the PCB loaded to MPs was not bioavailable. Furthermore, there was an increase in the bioaccumulation of PCB when it was associated with food particles [71]. Moreover, the lack of chemical affinity between the EC and NMPs (e.g., cadmium and PS-NMPs) prevented adsorption and did not influence MPs on bioaccumulation [70]. 

### 4.2. NMPs Influence the Bioaccumulation of Environmental Contaminants by Other Species (Protozoa, Bacteria, Macrophytes, Crab, and Bivalve)

The literature review on the influence of NMPs on the bioaccumulation of ECs in invertebrate and aquatic plant species came back with a total of 18 studies, from which nearly 50% used *Daphnia magna* as a model organism. The other species employed in the studies were *Euglena gracilis* (protozoa), *Streptomyces coelicolor* and *Escherichia coli* (bacteria), *Potamogeton crispus* and *Vallisneria denseserrulata* (macrophyte), *Vallisneria natans* (macrophyte), *Chlorella pyrenoidosa* (algae), *Eriocheir sinensis* (freshwater crab), and *Corbicula fluminea* (bivalve). In bacteria species, the presence of polystyrene nano- and microplastics (PS-NMPs) of varying sizes (100 nm and 1 μm) decreased the uptake and internalization of dibutyl phthalate (DBP), a plasticizer, by *S. coelicolor* [28] and Ag^+^ by *E. coli* [32]. The affinity of the hydrophobic compound DPB with the PS-NPs was higher, as detected by the decrease in DPB concentration in the bacteria culture media by up to 60% after the addition of NPs. Thus, the decrease in DPB internal concentration in bacteria occurred due to the low bioavailability of DPB in the media. 

Silver is known for its bactericidal action. However, when combined with polystyrene microplastics (PS-MPs) of 0.1 μm and 1 μm, the internalization of Ag^+^ by *E. coli* was significantly decreased [32] due to the presence of MPs in the media preventing Ag^+^ ions from entering the cell membrane. The chemical affinity of Ag^+^ to the polymers resulted in the adsorption of the metal ions to the MPs, thus reducing the free Ag^+^ in the bacteria culture media and, eventually, impairing Ag^+^ influx to the uptake sites of the bacterial cell. Similarly, this process can occur with other unicellular organisms, having cell walls, such as the green algae *C. pyrenoidosa* [33]. As the pore size of the algae cell wall varies between 5 and 20 nm [72] the carrier role of NMPs would only be observed for nanoplastics. For the free-living microorganism *E. gracilis* [27], the internal concentration of copper was not altered by the presence of PS-NMPs (75 nm and 1 μm). Although the authors previously demonstrated the uptake of MPs into the cell and chloroplast of the protozoa, the lack of influence of NMPs in the accumulation of Cu^2+^ was attributed to the adsorption of Cu^2+^ onto NMPs and sedimentation of Cu^2+^+NMPs in the exposure media, partly decreasing uptake. 

A loss of bioavailability probably caused by sedimentation of NMPs with adsorbed mercury (Hg) was also related as one of the reasons for the decrease in Hg content in the bivalve *Corbicula fluminea*. A bioconcentration factor (BCF) of 55 was obtained in the Hg-isolated exposure, whereas a BCF of 25 was found for combined exposure with MPs (unspecified polymer type) [30]. Moreover, the outcome of two studies that assessed metal uptake (Pb and Cd^2+^) in the submerged macrophyte *Vallisneria natans* [34] and *Potamogeton crispus* and *Vallisneria denseserrulata* [26] suggests no influence of microplastics in the accumulation of metals. In the study with *V. natans*, polyvinylchloride microplastics (PVC-MPs) and Cd^2+^ were mixed with sediment, possibly promoting the decreased availability of Cd to macrophytes by adsorption of Cd^2+^ to PVC and sediment particles. Although the authors argued that polymer particles with a positive charge can be attracted to the cellulose constituents of the plant cells, thus inducing mechanical damage, this process could also obstruct metal uptake sites. The two macrophytes showed species-specific responses to the combination of PS-MPs, and Pb. *P. crispus* accumulated more Pb in the presence of MPs, while the combined exposure did not affect the Pb concentration measured in *V. denseserrulata*. This again suggests that species-specific cellular mechanisms, morphology, and intrinsic means of metal uptake are core variables and deserve full consideration to understand the influence of NMPs on bioaccumulation. 

Experiments with the snail *B. aeruginosa* [29] exposed to Cd^2+^ and polystyrene nanoplastics (PS-NPs) via sediment and the crab *E. sinensis* [35] exposed to Pb and PS-MPs showed increased accumulated metals in both species, in the presence of NMPs. In the study with *Bellamya aeruginosa*, the authors also measured the free Cd^2+^ concentration in the porewater and in the visceral mass of the snail, presenting a correlation index of 0.94 between those two quantifications. Although the presence of PS-NPs increased the concentration of Cd^2+^ in snails by 45%, the increase was not associated with the carrier role of PS-NPs, but rather with the uptake of dissolved Cd^2+^ in the pore water. Pb levels measured in the hepatopancreas of *E. sinensis* increased in the presence of PS-MPs, but only after 14 and 21 days of exposure (on day 7, Pb levels were not altered by MPs). As the internalization of the PS-MPs (5 μm) was not confirmed by the authors, we cannot assume that the increase in bioaccumulation was a consequence of the carrier role.

Trophic transfer of methamphetamine was assessed from algae (*C. pyrenoidosa*) to snail (*Cipangopaludian cathayensis*) in the study of Qu et al. (2020) [73], where a comparison between exposure routes (water and dietary) in the accumulated methamphetamine by snails was also performed. By feeding snails with previously methamphetamine-exposed algae, the internal concentration of methamphetamine was double the amount detected in the water exposure alone, indicating an increase in the bioavailability of methamphetamine in the presence of algae, which played, in this scenario, a carrier role for both MPs and methamphetamine into the organs of the snail. 

### Daphnia magna

**Figure 5:**
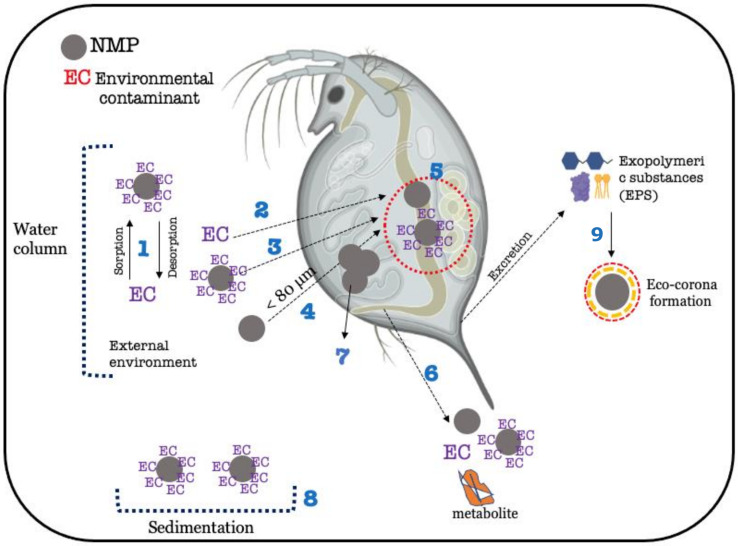
Simplified scheme of possible interactions between environmental contaminants (ECs) and NMPs in a freshwater environment and within the *Daphnia magna*. 1—Sorption/desorption of ECs onto NMPs. 2, 3, and 4—Uptake (via unselective filtering) of ECs (2), uptake of ECs adsorbed onto NMPs (3), and uptake of NMPs alone (4). 5—Sorption/desorption of ECs onto NMPs in the gastrointestinal tract after oral ingestion/filtration. Once in the gut, desorption of ECs from NMPs and metabolization of ECs may occur. NMP retention time is related to NMP size. 6—Excretion/egestion of NMPs, ECs, ECs adsorbed onto NMPs, and possible metabolites. 7—Obstruction of the filtering appendage by larger NMP particles that cannot be internalized (>80 μm). If the obstruction is caused by NMPs loaded with ECs, NMPs may act as a vehicle for EC delivery on site. 8—Sedimentation of NMPs loaded with ECs. In this case, the EC becomes less available, and uptake and bioaccumulation of the substance decrease. 9–Formation of eco-corona mediated by EPS (proteins, carbohydrates, and lipids).

As observed in fish, the increase in bioaccumulation induced by the presence of NMPs in *Daphnia magna* was mainly observed when nanoplastics were combined with ECs. In studies where the presence of NMPs increased bioaccumulation in *D. magna*, the size range of the NMPs used was from 50 nm to 900 nm, following previous studies suggesting that the maximum particle size internalized by *D. magna* is 80 μm [19]. However, there is a discrepancy in the nomenclature used by some authors who considered plastic particles larger than 100 nm as NPs [48]. For example, in the study performed by Monikh et al. (2020) [48], the smaller the particle used, the higher the bioconcentration of Ag^2+^ in *D. magna* exposed to PS-NPs (300 nm induced a BCF of 0.7, while 600 nm particles induced a BCF of 0.3). Similarly, *D. magna* accumulated more phenanthrene, a PAH, in the presence of PS-NPs (50 nm) than in its absence, whereas PS-MPs of 10 μm did not influence the amount of phenanthrene accumulated in *D. magna* [46]. Likewise, the internalization of pyrene, another PAH, was shown to be higher when pyrene was combined with PS-NMPs of 0.1–1.5 μm in comparison with bigger particles (10–60 μm and 60–230 μm) [17]. Interestingly, in the study of Lin et al. (2021) [17], the authors were able to disentangle the size of particles ingested (intestine) from the ones absorbed through dermal uptake and/or translocated through the intestinal barrier into other tissues. The latter only occurred for the 0.1–1.5 μm (100–1500 nm), while all sizes of PS-MPs used (up to 230 μm) were present in the intestine of *D. magna*. Recent studies outline that during the ecotoxicity testing of nanoplastics with *D. magna*, reduced toxicity is observed because of eco-corona formation [74]. The eco-corona is a layer of biological molecules adsorbed on the surface of the MNPs, which can modify their chemical features and interaction with biological barriers (Figure 5, process 8) [75]. The formation of the eco-corona is mediated by the presence of high amounts of natural organic matter (NOM) in solution, which adsorbs to the surface of the NMPs. In the case of *D. magna*, the excretion of exopolymeric substances (EPS, mainly carbohydrates, protein, and lipids) increases the amount of NOM in the media and favours the formation of a biomolecule-type layer outside the NMPs. Although eco-corona formation is not considered or verified in the bioaccumulation studies mentioned in the present review, it should be considered as one of the mechanisms responsible for the decrease in bioaccumulation of ECs in the presence of NMPs either due to sedimentation of NMPs with the eco-corona or loss of surface affinity between ECs and eco-corona-encapsulated NMPs [76].

Furthermore, the carrier role of NMPs was demonstrated by Jian et al. (2018) [50] by exposing *D. magna* to a series of concentrations of PS-NPs (100 nm) and keeping the concentration of PCB congeners in solution constant. With the application of a mass balance model, it was possible to observe an increase in the total uptake number of PS-NPs, from 75 to 259 ng per animal, as the concentration of PS-NPs increased from 2 to 20 mg/L, respectively. Concomitantly, the enhanced contributions of NP-bound PCBs to the total uptake amounts in *D. magna* at various NPs concentrations were 18–81%. As *D. magna* is a non-selective filtering feeder, with the increase in particle concentration, in this case, the encounter rate also increases, thereby eliciting higher feeding rates of NPs [11]. Moreover, PCBs with lower hydrophobicity conjugated with higher concentrations of PS-NPs generated higher internal concentrations of NP-bound PCBs in the organisms. Kim et al. (2017) [49] reported a concentration-dependent pattern of uptake and internalization of nickel (Ni) in the presence of PS-NPs (~200 nm). Up to 4 mg/L of Ni, treatments with PS-NPs present induced a higher accumulation of Ni by *D. magna*. However, at a Ni concentration of 5 mg/L, the accumulated Ni was half, relative to the Ni exposure alone. Although the authors do not discuss this result, it suggests a shift in the bioavailability of Ni stimulated by the increase in concentration, perhaps due to higher adsorption rates and sedimentation of Ni-loaded PS-NPs. 

## 5. Final Remarks and Identified Gaps

The present literature review aimed to answer whether NMPs influence the bioaccumulation of ECs by freshwater organisms. However, the answer to this question is more subjective than a simple matter of yes or no. We could gather evidence that major variables influence the outcome, the two most important ones being the size of the NMPs conjugated with the physiology of the organism—basically, whether the species under study can internalize the particles. Although some experimental procedures (e.g., particle concentration, exposure route) and particle features (size, polymer type) could affect the outcome of bioaccumulation, the organism’s physiology and developmental stages cannot be disregarded as a foremost factor that commands whether the presence of NMPs will influence the bioaccumulation of environmental contaminants [11]. A relationship between particle pre-treatment (e.g., pre-sorption or pre-incubation) and an increase in bioaccumulation could not be established, although it was previously assumed that a positive correlation would be noticed. The knowledge gaps identified in this literature review are presented below:

- Most studies performed particle pre-treatment (pre-incubation, pre-sorption) prior to exposure. However, no data are available to confirm whether particle pre-treatment will influence the results. This should be considered for further studies, especially in the cases where sorption occurs at optimal conditions (pH, Tº) different from the exposure condition (e.g., in the study of Yang et al. (2022) [35], PS-MPs were incubated with Pb 24 h prior to exposure at 25 °C, while exposure of the Pb-loaded MPs to snails occurred at 20–22 °C).
- Not all studies presented a bioconcentration or bioaccumulation factor (BCF/BAF). The presentation of those values and the size of the associated NMPs could improve the comparison of the potential for bioaccumulation of specific substances in combination with NMPs.
- The composition and shape of the polymers used were mainly represented by PS and spherical forms. Although it is difficult to produce nanoplastics of irregular shape, and many companies offer spherical nanoplastics to be used in the tests, perfect spherical NPs are most probably not found in the environment. That is because most nanoplastics are formed as the breakdown of larger plastic particles. Additionally, in the environment, the aging process of NMPs is extremely important, leading to the formation of eco-coronas (i.e., the presence of microorganisms and organic substances, such as proteins) and free radicals on the NMPs’ surface. Studies have shown how these processes affect the adsorption capacity of NMPs [74,75] and even affect the ecotoxicity of NMPs to aquatic organisms [76]. However, no studies on aged NMPs were found in this review. Future studies could use more realistic exposures, including irregularly shaped and aged NMPs.
- There is a lack of information regarding the influence of NMPs on the bioaccumulation of pesticides by freshwater species. Analytical techniques to quantify metal in animal tissue are cheaper and more accessible than the methodology required to quantify organic contaminants, such as the case of PPCPs and PAHs. On the other hand, given the hydrophobic nature of the NMP surface and, consequently, its higher affinity for non-polar substances, such as pharmaceuticals, pesticides, and other organic compounds, more studies should be conducted to test whether the presence of NMPs should facilitate the internalization and bioaccumulation of such substances in different species.

## Data Availability

The data that support the findings of this study are available from the corresponding author, (M.P.), upon reasonable request.

## Supplementary Materials

The following supporting information can be downloaded at: https://www.mdpi.com/article/10.3390/toxics11060474/s1, Table S1: Detailed information on the studies extracted from the literature review using the search strategy, inclusion and exclusion criteria described in the methods section.
