# Supplementary material for: Systematic Review of Nano- and Microplastics’ (NMP) Influence on the Bioaccumulation of Environmental Contaminants: Part II—Freshwater Organisms
Source: Toxics. 2023 May 23;11(6):474. doi: 10.3390/toxics11060474 (PMC10303264; doi:10.3390/toxics11060474)
Supplement: Supplementary file 1 [file toxics-11-00474-s001.zip › toxics-2356983-supplementary.pdf]

Table SI.1: Detailed information on the studies extracted from the literature review using the search strategy, inclusion and exclusion criteria described in the methods section.

n.a = not available	Study					Microplastic				Contaminant		Organisms		Outcomes
ID	Year	Media	Type	Exposure	MP treatment	Type	Size	Form	Material	Name	Chemical group	Species	Group	Influenced bioaccumulation?
3	2020	Freshwater	Lab experiment	water	no	NP	300-600 nm	spherical	PS	Ag	metals	*D. magna*	Zooplakton	yes, increased
3	2020	Freshwater	Lab experiment	water	no	NP	300-900 nm	spherical	PE	Ag	metals	*D. magna*	Zooplakton	yes, increased
31	2022	Freshwater	Lab experiment	water	no	NP	50 nm	spherical	PS	Al2O3, CeO2	nanoparticles	*D. rerio*	fish	yes, increased
41	2017	Freshwater	Lab experiment	water	no	NP	50 nm	spherical	PS	Bisphenol A	phenolics	*D. rerio*	fish	yes, increased
42	2022	Freshwater	Lab experiment	water	no	MP	5 um	spherical	PS	Cd	metals	*D. rerio*	fish	no
66	2018	Freshwater	Lab experiment	food	no	MP	100 to 500 µm	spherical	PE	PCBs	PCBs	*C.auratus*	fish	yes, decreased
74	2020	Freshwater	Lab experiment	water	pre-sorption	MP	48 um	spherical	PMMA	benzo(k)fluoranthene	PAHs	*D. magna and Chironomus riparius*	zooplankton	yes, increased (chironomus) and decreased (daphnia)
74	2020	Freshwater	Lab experiment	food (trophic transfer)	pre-incubation and then daphia ingestion	MP	48 um	spherical	PMMA	benzo(k)fluoranthene	PAHs	*D. rerio*	fish	no
83	2021	Freshwater	Lab experiment	water	no	MP	5 um	spherical	PS	sulfamethoxazole (SMX) and the β-blocker propranolol	PPCPs	*Oreochromis niloticusas*	fish	yes, increased
88	2022	Freshwater	Lab experiment	water	no	MP	100–150 µm	spherical	PS	Triclosan	PPCPs	*n.a*	fish	yes, increased
89	2019	Freshwater	Lab experiment	water	no	NP	100 nm	spherical	PS	PCBs	PCBs	*D. magna*	zooplankton	yes, increased
94	2017	Freshwater	in vitro	water	pre-incubation and no	MP	10–106 µm	spherical	PE	Ag	metals	*O. mykiss*	fish	no
95	2015	Freshwater	Lab experiment	water	pre-incubation and no	MP	10–106 µm	spherical	PE	Ag	metals	*D. rerio*	fish	yes, decreased (pre-sorption) and no (no pre-sorption)
97	2017	Freshwater	Lab experiment	water	no	NP	180-200 nm	spherical	PS	Ni	metals	*D. magna*	zooplankton	no

108	2022	Freshwater	Lab experiment	water	no	MP	50–70 µm	irregular	PS	Difenoconazole	pesticides	*D. rerio*	fish	yes, decreased
108	2022	Freshwater	Lab experiment	water	no	MP	9–10 µm	spherical	PS	Difenoconazole	pesticides	*D. rerio*	fish	no
110	2022	Freshwater	Lab experiment	water	pre-incubation	MP	180–430 µm	irregular	PE	PCBs	PCBs	*D. rerio*	fish	no
112	2022	Freshwater	Lab experiment	water	no	NP	75 nm	spherical	PS	Cu	metals	*E. gracilis*	protozoan	no
117	2021	Freshwater	Lab experiment	water	no	MP	0–1.5, 10–60, and 60–230 µm	irregular	PS	pyrene	PAHs	*D. magna*	zookplankton	yes, increased
119	2020	Freshwater	Lab experiment (kinetics)	water	pre-incubation	NP	117 nm	spherical	PS	acenaphthene, fluorene, phenanthrene, anthracene, fluoranthene and pyrene	PAHs	*D. magna*	zookplankton	yes, decreased
124	2022	Freshwater	Lab experiment	water	no	NP	100nm	spherical	PS	dibutyl phthalate	alkylphenols	*S.coelicolor*	bacteria	yes, decreased
127	2018	Freshwater	Lab experiment	water	pre-incubation	MP	5 um	spherical	PS	Cd	metals	*D. rerio*	fish	yes, increased
128	2022	Freshwater	Lab experiment	water	pre-incubation	NP	80 nm	spherical	PS	Cd	metals	*B. aeruginosa*	gastropod	yes, increased
130	2016	Freshwater	Lab experiment	water	no	NP	50 nm	spherical	PS	phenanthrene	PAHs	*D. magna*	zooplankton	yes, increased
130	2016	Freshwater	Lab experiment	water	no	MP	10 um	spherical	PS	phenanthrene	PAHs	*D. magna*	zooplankton	no
139	2021	Freshwater	Lab experiment	water	pre-sorption	MP	250-500nm	irregular	PE	benzophenone-3	benzophenone-3	*D. magna*	zooplankton	yes, increased
145	2022	Freshwater	Lab experiment	water	pre-incubation	MP	3 um	spherical	PS	Pb	metals	*P. crispus & V. denseserrulata*	macrophytes/plants	no
147	2018	Freshwater	Lab experiment	water	no	MP	1 and 5 µm	spherical	nd	Hg	metals	*C. fluminea*	bivalve	yes, decreased
154	2019	Freshwater	Lab experiment (kinetics)	water	pre-incubation	NP	100nm	spherical	PS	Cu	metals	*D. rerio*	fish	yes, increased
154	2019	Freshwater	Lab experiment (kinetics)	water	pre-incubation	MP	20 um	spherical	PS	Cu	metals	*D. rerio*	fish	yes, increased
156	2020	Freshwater	Lab experiment	water and food	no	NP	700 nm	spherical	PS	racemic methamphetamine	PPCPs	*C. cathayensis*	snail	yes, increased and no
157	2019	Freshwater	Lab experiment	water	no	MP	<10 µm	spherical	PVC	venlafaxine and O-desthylvenlafaxine	PPCPs	*M. anguillicaudatus*	Fish	yes, increased

171	2021	Freshwater	Lab experiment	water	no	MP	1-5 um	spherical	nd	Cu	metals	*D. rerio*	fish	no
175	2022	Freshwater	Lab experiment	water	pre-incubation	MP	150 µm	irregular	PE	chlorpyrifos and hexachlorobenzene	pesticides	*D. rerio*	fish	yes, decreased and no
180	2021	Freshwater	Lab experiment	water	pre-incubation	MP	1–15 µm	irregular	PE, PP, PVC	triclosan	PPCPs	*D. rerio*	fish	yes, increased and decreased
189	2020	Freshwater	Lab experiment	water	no	MP	1.0 µm	spherical	PS	Ag	metals	*E. coli*	bacteria	yes, decreased
189	2020	Freshwater	Lab experiment	water	no	NP	0.1 µm	spherical	PS	Ag	metals	*E. coli*	bacteria	yes, decreased
213	2020	Freshwater	Lab experiment	water	no	NP	600 nm	spherical	PS	ibuprofen	PPCPs	*C. pyrenoidosa*	phytoplankton	no
223	2021	Freshwater	Lab experiment	sediment (Freshwater)	pre-incubation	MP	n.a	irregular	PVC	Cd	metals	*V. natans*	Plant	no
227	2018	Freshwater	Lab experiment	water	pre-incubation	MP	32–40 µm	spherical	PS	Cd	metals	*S. aequifasciatus*	fish	yes, decreased
235	2021	Freshwater	Lab experiment	water	pre-incubation	MP	150 um	irregular	PS	Phenanthrene	PAHs	*D. rerio*	fish	yes, increased
238	2020	Freshwater	Lab experiment	water	pre-incubation	MP	5 um	spherical	PS	F-53B	PFASs	*D. rerio*	fish	yes, decreased
240	2022	Freshwater	Lab experiment	water	pre-incubation	MP	5 um	spherical	PS	Pb	metals	*E. sinensis*	crab	yes, increased
243	2021	Freshwater	Lab experiment	water	no	MP	5 um	spherical	PS	Tetracycline	PPCPs	*C. carpio*	fish	yes, decreased
244	2022	Freshwater	Lab experiment	water	no	NP	100 nm	spherical	PS	Cu	metals	*O. niloticus*	fish	yes, increased
245	2021	Freshwater	Lab experiment	water	pre-sorption	MP	100–150 µm	spherical	PS	9-NAnt	PAHs	*D. rerio*	fish	yes, increased and decreased
248	2019	Freshwater	Lab experiment	water	no	NP	100 nm	spherical	PS	Roxithromycin	PPCPs	*O. niloticus*	fish	yes, increased and decreased
251	2022	Freshwater	Lab experiment	water	no	MP	5um	spherical	PS	ZnO NPs, ZnSO4	nanoparticles, metal	*D. rerio*	fish	yes, increased and decreased
253	2021	Freshwater	Lab experiment	water	pre-incubation	NP	100 nm	spherical	PS	ethylhexyl salicylate	PPCPs	*D. rerio*	fish	yes, increased
254	2022	Freshwater	Lab experiment	water	pre-incubation	NP	101 nm	spherical	PS		PPCPs	*D. rerio*	fish	yes, increased
257	2022	Freshwater	Lab experiment	water	no	MP	5 um	spherical	PS	MeHg	metals	*D. rerio*	fish	yes, increased
